# Intrinsically distinct hole and electron transport in conjugated polymers controlled by intra and intermolecular interactions

**DOI:** 10.1038/s41467-019-13155-9

**Published:** 2019-11-19

**Authors:** Giuseppina Pace, Ilaria Bargigia, Yong-Young Noh, Carlos Silva, Mario Caironi

**Affiliations:** 1 Center for Nano Science and Technology@PoliMi, Istituto Italiano di Tecnologia, Via Pascoli 70/3, 20133 Milano, Italy; 2 School of Physics, School of Chemistry and Biochemistry, Georgia Institute of Technology, Atlanta, Georgia, 901 Atlantic Drive, Atlanta, GA 30332-0400 USA; 3 Department of Chemical Engineering, Pohang University of Science and Technology, 77 Cheongam-Ro, Nam-Gu, Pohang, 37673 Republic of Korea

**Keywords:** Electronic properties and materials, Electronic devices

## Abstract

It is still a matter of controversy whether the relative difference in hole and electron transport in solution-processed organic semiconductors is either due to intrinsic properties linked to chemical and solid-state structure or to extrinsic factors, as device architecture. We here isolate the intrinsic factors affecting either electron or hole transport within the same film microstructure of a model copolymer semiconductor. Relatively, holes predominantly bleach inter-chain interactions with H-type electronic coupling character, while electrons’ relaxation more strongly involves intra-chain interactions with J-type character. Holes and electrons mobility correlates with the presence of a charge transfer state, while their ratio is a function of the relative content of intra- and inter-molecular interactions. Such fundamental observation, revealing the specific role of the ground-state intra- and inter-molecular coupling in selectively assisting charge transport, allows predicting a more favorable hole or electron transport already from screening the polymer film ground state optical properties.

## Abstract (web summary)

Determining the structure-property relationship responsible for charge transport in organic semiconductors remains a challenge. Here, the authors, through spectroscopic & microscopic studies on organic transistors, report the intrinsic factors that affect charge transport in polymer semiconductors.

## Introduction

Organic semiconductors have been extensively investigated in the past few decades, particularly in view of their applications in new lightweight and flexible microelectronics, optoelectronic, and sensing devices^1,2^. Solution-processable organic semiconductors have enabled the formulation of printable inks extending their application in large-area and cost-effective electronics^3–5^.

Recent progresses have led to the synthesis of a wide range of homopolymers and donor–acceptor (D–A) copolymers^6^, showing high charge carrier mobilities, in the range of 1–10 cm^2^/V s^7^. Among ambipolar copolymers, diketopyrrole–pyrrole (DPP) derivatives^8–11^ have shown field-effect mobilities consistently higher than 1 cm^2^/V s for both carriers^12–14^. High mobilities are favored by mesoscopically ordered packings favored by an improved backbone planarity^15–17^, higher molecular weight^18^, as well as side chain engineering^19,20^. It has been proposed that longer polymer chains can favor charge transport by enhancing the interconnectivity between ordered polymer phases and amorphous-like ones^17^. An important step forward to improving charge transport properties is the introduction of techniques that favor the unidirectional alignment of polymer chains^21–23^. There are consistent proofs showing that, in organic field-effect transistors (OFETs), the current flowing within the transistor channel strongly improves when polymer chains are anisotropically aligned perpendicularly to the source and drain electrodes, demonstrating the favorable charge transport along the polymer chain^18,21,22,24^.

In spite of the great advances made in the field, it is still a truly brain teaser whether holes and electrons can be equally well transported within the same material microstructure once they are injected from the electrodes, or if there are extrinsic effects favoring either the one species or the other. Though interfacial phenomena can play a major role in unbalancing the charge transport of the two carriers, the tickling question regards the influence of polymer chain chemical composition and solid-state microstructure on a preferential hole or electron transport.

In their work, Bredas et al. investigated, from a theoretical point of view, the role played by parameters such as chemical composition, backbone planarity, and extension of intramolecular and intermolecular π–π conjugation, in selectively affecting hole and electron-hopping transport^25^. They particularly gathered attention on the strong impact played by the relative distance and orientation of neighboring chromophores, and on the superposition of their outer molecular orbitals. Very small distortion, of the order of a fraction of an angstrom, can determine a wide variation  in the chromophores’ electronic coupling, having an impact on holes and/or electrons charge transfer (CT) probability. Such information is clearly embedded in the semiconductor ground-state spectra at the solid state, but until now, no keys have been provided to obtain hole and/or electron mobility insights from such spectra, a clear limitation to their predictive power. Nevertheless, such predictability would be fundamental for identifying those design principles that can boost further the desired unipolar or ambipolar transport properties of new polymers.

Ideal candidates for addressing the above points and simultaneously studying the intrinsic nature of hole and electron transport are ambipolar polymer devices where both charges can be transported similarly well. Between polymers showing good balance between hole and electron mobility, we selected a DPP derivative (diketopyrrolopyrrole-thieno[3,2-b]thiophene, DPPT-TT), where hole and electron transport can be investigated within the same device, while keeping the same chemical composition, thin-film morphology, and device architecture. Here, through charge-modulation spectroscopy and microscopy (CMS and CMM) studies on operating OFETs^26^, as well as polarized UV–Vis and transient absorption (TA) spectroscopy acquired on thin DPP polymer films, we highlight for the first time the preferential ground-state molecular interactions that can selectively favor either hole or electron transport in a D–A copolymer semiconductor. A first evidence of the different spectral signature of holes and electron polarons found in the charge absorption region was presented by Kathib et al.^27,28^. However, the two polaron signatures appeared as a broad band in the near-infrared (NIR) region, making it difficult to provide information on their inter- or intrachain nature. Here, by comparing polarization- dependent ground-state absorption spectra of anisotropically aligned polymer films with CMS bleaching spectra, we observe that holes and electrons selectively bleach different features of the ground-state absorption. We found that specific inter- and intramolecular interactions can favor either electron or hole transport, respectively.

The theoretical framework describing the electronic coupling between repeating chromophores in conjugated polymers that is at the base of such inter- and intramolecular interactions was proposed by Prof. Spano^29^. Specifically, J-type electronic coupling occurs between covalently linked chromophores within the polymer chain, while H-type coupling involves the electronic coupling between cofacial chromophore units of neighboring polymer chains.

In this work, we show that the relative strength of intrachain and interchain electronic coupling, identifying a more J- or H-type coupling, respectively, sizes the ratio between electron and hole relative mobility. This correlation between carriers’ mobility and spectral features makes it possible to predict from simple optical absorption measurements the balance between hole and electron transport. While no absolute mobility values can be extracted from absorption spectra due to the lack of a universal quantitative model, we show that the spectral variations observed on different polymer films, including the ones reported here and in the previous literature, contain the necessary information for predicting the expected mobility trend in the investigated polymer films.

## Results

### Aligned polymer films morphological and optical anisotropy

We selected a well-known DPP derivative, DPPT-TT^30,31^. As for other high-molecular-weight polymers owning thermotropic and/or lyotropic liquid crystal properties^14,32^, DPPT-TT can be easily aligned, allowing a high control on its film morphology, improved charge transport, and electrical anisotropy^18,24,31,33,34^. In this work, we employed the off-center spin-coating technique to mechanically force the polymer chains alignment along the centrifugal force direction established during spinning^34^. In such films, DPPT-TT polymer chains have an edge-on backbone orientation and are aligned along the radial direction, as previously demonstrated^11^. When DPPT-TT polymer chains are aligned perpendicularly to the source and drain electrodes of a field-effect transistor, a ~40 times higher hole mobility can be measured with respect to the case where the chains are aligned parallel to the channel^31^.

**Fig. 1:**
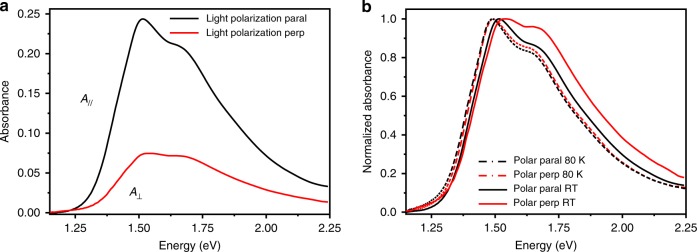
UV–Vis and NIR absorption spectra. **a** Polarization-dependent spectra of an off-center spin coated, aligned DPPT-TT film (from chlorobenzene solution, 10 mg/ml). **b** Normalized data of the polarization- dependent spectra at room temperature and at 80 K (trichlorobenzene solution, 10 mg/ml). The temperature dependence is reversible upon cycling (Supplementary Fig. 2). Light polarized parallel (polar paral, *A*_//_) or perpendicular to the chain alignment direction (polar perp, *A*_⊥_)

Further evidence of the degree of polymer chains alignment is found in the angular dependence of the polarized absorption spectra (Fig. 1a, Supplementary Fig. 1). The maximum optical absorption occurs for an incident polarized light parallel to the chain alignment direction (*A*_//_, parallel component of the absorption spectrum), while the minimum is observed when the light is polarized perpendicular to the chains (*A*_⊥_, orthogonal component of the absorption spectrum). Two main peaks are present in both spectra, located at around 1.51 and 1.67 eV for *A*_//_, and only slightly blue shifted in *A*_⊥,_ similarly to what was reported for DPPT-TT films aligned employing solution shearing^30^ or slot die coatings^12^. For our films, the dichroic ratio (*D*), defined as *A*_//_/*A*_⊥_, is ∼2.6 at 1.51 eV (820 nm, Fig. 1a)^35–37^. The optical anisotropy data, along with previous 2D-GIXRD findings^38^, clearly indicate the presence of an electronic transition dipole moment (TDM) aligned along the polymer chain main axis.

A weak shoulder located at ∼1.45 eV, more pronounced in *A*_⊥_, can be observed (Supplementary Figs. 3 and Supplementary Note 1). Since it is not possible to make any assignment of such red shoulder only on the basis of the absorption spectra, we further investigated its nature via TA studies.

**Fig. 2:**
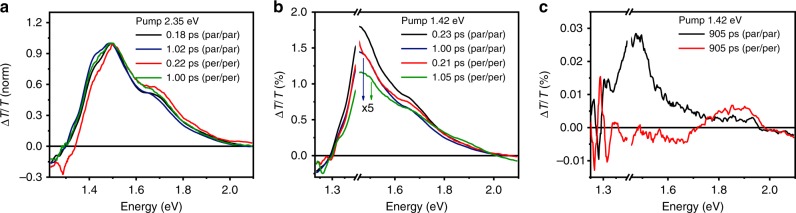
Polarized transient absorption spectra (TA). Pump/probe light beams polarized either parallel (paral/paral) or perpendicular (perp/perp) to the polymer chain main axis. **a** TA spectra acquired with a pump wavelength of 2.35 eV at 0.22 and 1 ps. **b** TA spectra acquired with a pump wavelength of 1.42 eV at (**b**) 0.23 and 1.00 ps; (**c**) 905 ps

Due to their highest dichroic ratio (∼0.59), TA spectra were acquired on films spun from chloronaphthalene (10 mg/ml) either exciting above the bandgap at 2.35 eV (Fig. 2a) or at the red edge of the absorption spectra (pump at 1.42 eV, Fig. 2b, c). The pump and probe light beams were polarized either parallel to the polymer chain main axis (pump/probe, paral/paral) or orthogonal (pump/probe, perp/perp). When exciting at high energy, the red shoulder at 1.42 eV appears more pronounced at longer timescale (~1 ps) due to its slower decay with respect to the rest of the ground-state bleaching (Supplementary Fig. 4). By exciting at the red edge of the absorption spectra (Fig. 2b), we can observe that such excited state is directly accessible from the ground state. The time decay of TA acquired with paral/paral excitation shows a very fast initial component that dies off within 10 ps, followed by a very long-lived dynamic (>1 ns; Supplementary Fig. 4). These proofs strongly suggest that the red shoulder in the ground-state absorption, appearing at ∼1.45 eV and associated with a longer-lived exciton, has a more marked CT character. In the following, we will refer to such state as a CT exciton, in analogy to previous reports on similar features observed in other D–A low bandgap copolymers^39–43^. From the time decay of the TA spectra acquired with perp/perp excitations, it can be clearly observed that higher-energy peaks appearing at 1.51 and 1.67 eV are different in nature, being associated with shorter-lived excited state (<10 ps, Supplementary Fig. 4)^44–46^. A strong anisotropy of the TA spectra (paral/paral vs. perp/perp) can be observed also at longer timescale (Fig. 2c), where distinct relaxed excited states are present, demonstrating the very different energy landscape experienced by the excitons generated when the pump beam is either polarized parallel to the main chain axis or perpendicular to it.

Therefore, we identified at least two distinct electronic transitions in the linear absorption spectrum, one at ∼1.45 eV dominating the red shoulder that we assigned to a CT exciton, and the other characterizing the remaining visible region. The maximum TA signal found for the CT band is recorded when both pump and probe beams are polarized along the polymer backbone alignment direction, indicating that the TDM of the CT state (TDM_CT_) lays parallel to the polymer chain (Fig. 2b). The low-energy feature at 1.51 eV of the ground-state absorption is assigned to the 0–0 vibronic component of the π−π* transition and the 1.67 eV feature to the 0–1 one, in agreement with previous assignments in the literature for similar DPP derivatives^47,48^.

The vibronic features of the π–π* transition often observed in semiconducting polymers, can be mostly described within the framework of J- and H-type interactions^49^. J-type interactions identify intrachain, through bond excitonic coupling, arising from a head-to-tail orientation of the chromophores placed along the polymer chain. H-type interactions refer to interchain excitonic coupling between face-to-face arranged chromophores belonging to neighboring polymer chains. The coexistence of H- and J-type interactions in disordered polymer films has been shown experimentally, and theoretically described^50–52^, for polydiacetylene (PDA)^52^ and poly[2-methoxy-5-(2-ethylhexyloxy)-1,4-phenylenevinylene] (MEH-PPV)^51^. In conjugated molecular crystals^42,53^, two different ground states with orthogonal TDMs having a distinct more prominent J or H character have been identified, and our observation well aligns with this conclusion that therefore extends also to highly ordered polymer films. H- and J-type interactions are characterized by distinctive signatures in the absorption and emission spectra and by a strongly different temperature dependence^47–51^. The relative strength of the progressing vibronic features of the π−π* optical transition reveals which is the more prevalent interaction (*I*_0–0_/*I*_0–1_, where *I*_0–0_ is the intensity of the 0–0 feature, and *I*_0–1_ of the 0–1). This parameter is proportional to the Huang–Rhys factor (*λ*^2^, 1/*λ*^2^ ∝ *I*_0–0_/*I*_0–1_), which quantifies the coupling between an electronic transition and a phonon mode^54^. In particular, a decrease (increase) in the *I*_0–0_/*I*_0–1_ ratio, and a spectral blue (red) shift indicates the incurring presence of H- (J)-type interactions^29,49^. We observe that *I*_0–0_/*I*_0–1_ is equal to 1.19 in *A*_//_, while it reduces to 1.08 in *A*_⊥_ (Supplementary Table 1). As a reference, we extracted such ratio factor also in DPPT-TT solutions at low concentration, where single chains and mostly J-type interactions are present (*I*_0–0_/*I*_0–1 _= 1.24, Supplementary Fig. 5 and Supplementary Table 1). Therefore, J-type interactions are prevalent in *A*_//_, reflecting their intrachain nature along the polymer chain, while a stronger contribution of H-type interactions is present in *A*_⊥_, as expected from their interchain nature. Proofs of the copresence of two distinct H- and J-type optical transition dipoles with an almost orthogonal orientation have been previously found in small-molecule crystals^55,56^. Such hybrid assignment of coexisting J- and H-type interactions within the same molecular packing and the presence of orthogonal excitons has not been studied to the same extent in polymer films; nevertheless, there is a consistent number of reports identifying the coexistence of H- and J-type interactions also in this case^50,55^.

While photoluminescence (PL) is typically adopted to confirm the nature of the intra- and intermolecular interactions, this is not viable for DPPT-TT, due to its extremely low PL quantum yield in solution, which becomes even lower in solid-state films^10^. We therefore resorted to temperature-dependent absorption measurements. The temperature-dependent spectral changes reported in Fig. 1b are completely reversible (Supplementary Fig. 2). At room temperature (RT), for *A*_⊥_ we observe that *I*_0–0_/*I*_0–1 _= 1.06 (Supplementary Table 1). By lowering the temperature down to 80 K, the spectra show a ∼40 meV red shift of the *I*_0–0_ peak and an increase in the *I*_0–0_/*I*_0–1_ relative peak intensity (*I*_0–0_/*I*_0–1 _= 1.20), indicating an increasing J-type character at lowering the temperature. Such H to J transition with temperature was previously observed in poly(3-hexylthiophene) (P3HT)^52,54^ and MEH-PPV^36,51^, implying that the overall photophysical response of such semiconducting polymers arises from the presence of both kinds of interactions. The raising of the J-type feature at lower temperature can be rationalized in terms of reduced thermal disorder favoring a backbone planarization^27,57^.

### Charge-modulation investigation in field-effect transistors

We here investigate in detail the specific spectral features of holes and electrons transported in DPPT-TT OFETs, by means of CMS and CMM performed on devices where the polymer chains are aligned perpendicularly to the source and drain electrodes^31^. The ambipolar electrical characteristics are reported in Supplementary Fig. 6. CMS is a lock-in-based technique that measures the variation in the optical transmission (Δ*T*), normalized to the total transmission (Δ*T*/*T*), of a semiconducting polymer film embedded in an operating OFET. The optical variation is caused by the frequency modulation of the accumulated charge carrier density due to the AC gate voltage superimposed to the DC bias. Only charges that are effectively contributing to the charge transport within the timeframe of the modulation frequency are probed, excluding deeply trapped, non-mobile states^58,59^. Therefore, CMS selectively probes those chromophores carrying a mobile charge in the few molecular layers lying below the dielectric interface where the charge accumulation takes place^60–62^. A positive CMS signal (Δ*T*/*T* > 0), named bleaching, is a signature of an increased light transmission associated with a reduction of the density of absorbing neutral polymer segments, a consequence of the presence of mobile charges. Therefore, the bleaching region carries a direct spectral signature of the intra- and intermolecular environment sensed by the charge. A negative CMS signal (Δ*T*/*T* < 0) evidences the presence of new optical transitions generated by the absorption from the charged chromophores.

**Fig. 3:**
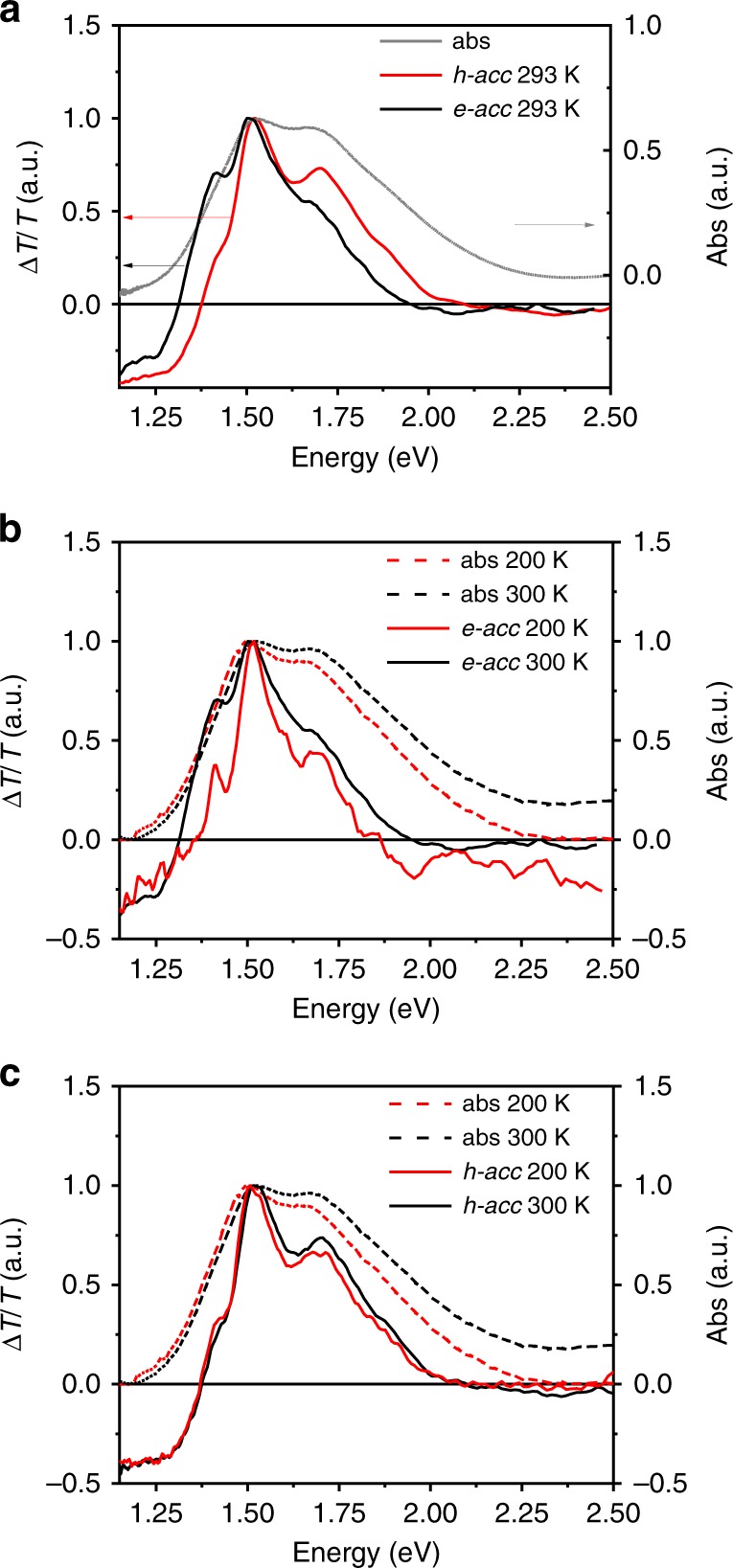
Normalized temperature-dependent CMS (continuous line) and absorption spectra (dashed line). **a** Comparison between absorption spectra, *h-acc* and *e-acc* CMS spectra acquired at room temperature. **b** Comparison between absorption spectra and *e-acc* CMS spectra acquired at 300 and 200 K. **c** Comparison between absorption spectra and *h-acc* CMS spectra acquired at 300 and 200 K (*e-acc*: *V*_g_ = 30 V; *V*_pp_ = 40 ± 20 V; *h-acc*: *V*_g _= −30 V; *V*_pp_ = 40 ± 20 V). The selected temperatures allow comparing the CMS spectra without incurring in the presence of lineshapes due to the interference from electroabsorption features (see Supplementary Figs. 7 and 8)

In Fig. 3 we report the CMS spectra acquired under hole and electron-accumulation regimes, indicated in the following as *h-acc* and *e-acc*, respectively. Below 1.3 eV, a broad charge absorption band appears in both spectra, while the 1.3–2-eV range is dominated by the ground-state bleaching. Figure 3a shows the correspondence of the CMS bleaching peaks with the ones observed in the ground-state absorption spectra. The finer structure found in the bleaching region for both *e-* and *h-acc* CMS spectra with respect to the UV–Vis absorption is a typical feature found in CMS spectra of different organic semiconductors, indicating that mobile charges preferentially populate regions of higher structural order^58,59^.

For both carriers, we distinguish three bleaching peaks appearing at 1.42, 1.51, and 1.71 eV. The relative intensity of the 1.51 and 1.71 eV features, corresponding, respectively, to the 0–0 and 0–1 vibronic replica, is strongly different in the two cases. We further notice that the red shoulder at 1.42 eV, corresponding to the CT state, is much stronger in the *e-acc* spectrum than in the *h-acc* one, the latest being consistently blue shifted.

Very importantly, such observation of strongly different relative intensities of the main bleaching peaks in CMS spectra under *h-acc* and *e-acc* clearly indicates that holes and electrons selectively bleach different spectral components of the overall thin film ground-state absorption.

By considering the relative intensity of the 1.51 and 1.71 eV features, we observe a stronger H-type character (*I*_0–0_/*I*_0–1_ = 1.26) in the bleaching region under *h-acc*, while under *e-acc*, the bleaching reveals the prevailing J-type coupling (*I*_0–0_/*I*_0–1_ = 1.42). It is not possible to establish a well-defined cutoff value for such peak ratio that would enable us to identify uniquely pure J-type interactions and pure H-type interactions since in polymer films they are concomitantly present. However, a relative comparison will enable us to follow the relative content variation in H- or J-type coupling. To corroborate this evidence, in Fig. 3b, c we overlapped the non-polarized ground-state temperature-dependent absorption spectra with the corresponding temperature-dependent *e-acc* and *h-acc* CMS spectra. The temperature dependence of the CMS *I*_0–0_/*I*_0–1_ ratio confirms the increasing J character of the molecular interactions involved in the charge relaxation at lowering the temperature^29,49,54,63^. A weak red shift of the 0–0 peak from 1.52 to 1.50 eV in *h-acc* spectra can also be observed (Fig. 3c), confirming the transition to increasing J-type character^63^.

The above experimental data represent the first fundamental evidence that holes and electrons relax within the polymer film selectively perturbing different intra- and intermolecular interactions: relatively, holes mostly bleach H-type intermolecular interactions, while electrons preferentially bleach J-type intramolecular interactions. This fundamental finding highlights their intrinsic different transport characteristics.

Moreover, in the *e-acc* CMS, the relative intensity of the 0–0 peak with respect to the CT peak strongly increases at lowering the temperature. Since the TDM_CT_ state is aligned parallel to the polymer backbone, as evidenced by TA measurements, this might imply a stronger coupling between J-type interactions and CT states when an electron relaxes on the chain. At lowering the temperature, owing to the increased J-type interactions, a strong temperature dependence of the CT peak is indeed observed only in the *e-acc* CMS spectra, contrarily to the *h-acc* ones where the CT peak is already weak at RT.

By coupling CMS with an optical microscope, it is possible to spatially resolve, with a submicrometer resolution, the mobile charge signal within the OFET channel (charge modulation microscopy, CMM)^26,31,64–66^. We also acquired local CMS spectra within the active channel, decoupling the CMS channel signal from eventual spurious effects, such as electroabsorption at the injecting electrodes^67^. The consistency of the spectral features found in the macroscopic CMS, acquired on the entire device, with the local CMS spectra demonstrates the absence of such interfering spurious effects (Supplementary Figs. 9 and 10).

**Fig. 4:**
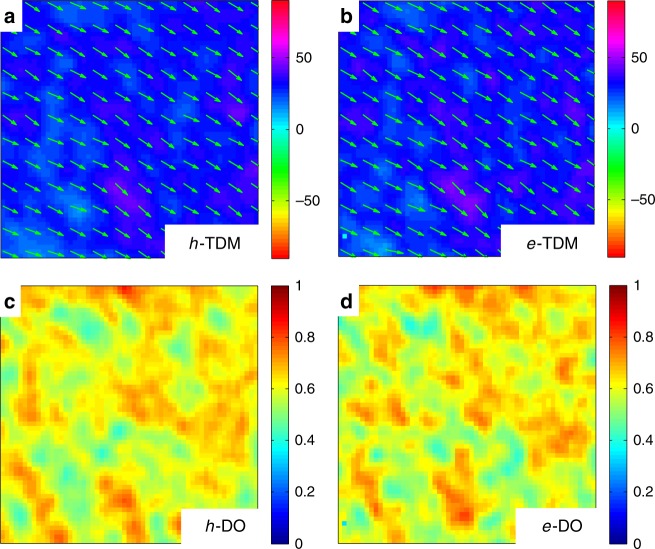
CMM maps of the active channel probed at 1.18 eV, within the photoinduced charge absorption region. (15 μm × 15 μm) Source and drain electrodes are located at the left and right side of the maps. **a**, **b** Charge transition dipole moment (TDM_charge_) maps acquired under *h-acc* and *e-acc*, showing the angular orientation of the anisotropic component of the charge modulation signal. Green arrows indicate that the TDM_holes_ and TDM_electrons_ mostly distribute along the polymer chains alignment direction. **c**, **d** Maps acquired under *h-acc* and *e-acc*, showing the local degree of order (DO), quantifying the weight of the anisotropic component of the charge modulation signal with respect to the isotropic component. Probing conditions: for *e-acc*, *V*_g_ = 30 V, *V*_pp_ = 40 ± 20 V; for *h-acc*, *V*_g_ = − 30 V; *V*_pp_ = 40 ± 20 V

Light polarization-dependent CMM allows us to extract angular maps indicating the orientation of the optical TDM at a specific wavelength, and the degree of order (DO), quantifying the fraction of charge-modulated signal that originates from anisotropically oriented TDMs (Supplementary Note 2)^65^. Figure 4 shows the angular and DO maps of the charge TDM (TDM_charge_) acquired within the OFET channel under *e-acc* and *h-acc* regimes, respectively. At the chosen probing wavelength (1.18 eV), only the broad charge-induced absorption signal is detected, no ground-state absorption exists, and the anisotropic contribution to the angular map is associated only to the anisotropy of TDM_charge_. We further confirm that for DPPT-TT transistors the TDM_charge_ is aligned along the polymer chain main axis, providing a direct information on the preferential orientation of the conjugated segments probed by the charge^31,66^. TDM_charge_ angular maps found for holes and electrons are very similar, showing that in both cases TDM_charge_ are predominantly aligned along the chain alignment direction (Fig. 4a, b).

**Fig. 5:**
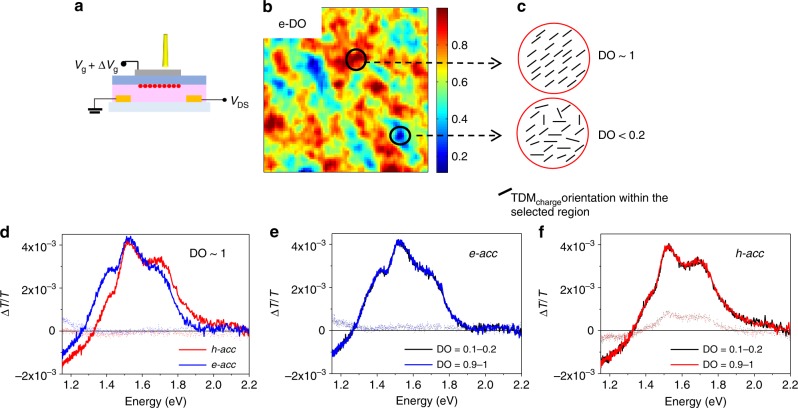
Comparing local CMS spectra acquired in regions with DO ≈ 1 and DO < 0.2. Top panel: **a** scheme of the OFET device under local CMS light probe. **b** *e-acc* DO map (15 μm ×15 μm). **c** Schematics of charge transition dipole moment alignment (TDM): black circles indicate regions of DO ≈ 1 and DO < 0.2, whose TDM vector distribution is sketched in the drawing on the right side. **d** *h-acc* and *e-acc* spectra acquired in an area with DO ≈ 1. **e** *e-acc* local CMS spectra acquired in regions with, respectively, DO ≈ 1 and DO ≈ 0.2. f *h-acc* local CMS spectra acquired in the region with, respectively, DO ≈ 1 and DO ≈ 0.2 (OFET electron accumulation, *e-acc*: *V*_g _= 30 V; *V*_pp_ = 40 ± 20 V; hole accumulation, *h-acc*: *V*_g _= −30 V; *V*_pp_ = 40 ± 20 V; dashed lines, out-of-phase signal; degree of order, DO)

The recorded DO maps (Fig. 4c, d) show that in both accumulation regimes, there are regions with high degree of directional order (in the range from 0.7 to 1) widely present over the entire scanned area. Regions with DO ≈1 refer to areas where the entire charge modulation signal arises from very well-aligned TDM_charge_. Only in very limited regions, the TDM_charge_ is more randomly distributed (DO < 0.2) (Fig. 5). We found a perfect overlap of the CMS spectra collected in regions with DO ≈ 1 and DO < 0.2 for both accumulation regimes (Fig. 5a–c). Such important evidence demonstrates that the nature of the charged state is not dependent upon the degree of the directional order of a specific region. The spectra found under *h-acc* and *e-acc* must therefore arise from the same ordered aggregates, which are present throughout the film independently from the local concentration of aligned chains and that are selectively probed by mobile charge.

Therefore, the preferential bleaching of inter- or intramolecular interactions occurring during either electron or hole transport is uniquely selected by the nature of the mobile charge and not by the specific local morphology of the film.

### Correlating transport properties to ground-state transitions

We have so far demonstrated the different nature of the molecular interactions affected by holes and electrons when relaxing on a polymer site involved in transport. We now exploit such fundamental insight to show that it is possible to gain information on hole and electron transport already from the analysis of the ground-state transitions. The lack of a generally applicable theory modeling organic semiconductors absorption bands, which could enable the precise extrapolation of the different overlapping vibronic peaks, does not allow us to collect from those spectra absolute values that can be directly compared with mobility values. Nevertheless, it is possible to take into account relative variations occurring along different polymer films absorption spectra and correlate those variations with mobility changes.

We here correlate the solvent dependence of UV–Vis absorption spectra in DPPT-TT films, with their hole and electron mobility. We recall that the degree of chain alignment, and the film dichroic ratio, achievable with off-center spin coating is dependent on the solvent adopted in the DPPT-TT solutions, being the highest in films spun from chloronaphthalene (cn), followed by films spun from trichlorobenzene (tcb), chloroform (cf), chlorobenzene (cb), and toluene (tol)^31^. It was previously shown that hole mobility follows a similar trend, because of the positive effect of the degree of aligned chains on the charge mobility. We note here that also electron mobility, despite being an order of magnitude lower, increases from toluene to chloronaphthalene in the same order as found for hole mobility (Supplementary Figs. 11 and 12). Interestingly, from the UV–Vis absorption spectra it is possible to observe that also the CT contribution to the ground-state absorption follows the same solvent dependence. Therefore, the highest mobility, both for holes and electrons, is reached in films spun from chloronaphthalene, for which both the dichroic ratio and the ground CT state are higher than what was found for the other solvents. The latter is in line with the observation of a cross-coupling between the CT and the π–π* transitions found in the *e-acc* and *h-acc* CMS, suggesting that an increased content of aligned ordered aggregates in the films contributes to improve the mobility, through an increased uniaxial transport and CT state content.

Since the presence of either a hole or an electron along the polymer chain selectively bleaches H-type and J-type interactions, respectively, the question is if this different behavior of the charge surrounding can be interpreted in terms of a more or less favorable transport for each of those charges. In order to provide an answer to such question, we considered the relative ratio between electrons (*μ*_e_) and holes (*μ*_h_) mobility to assess the influence of those H- and J-type interactions on their relative transport.

**Fig. 6:**
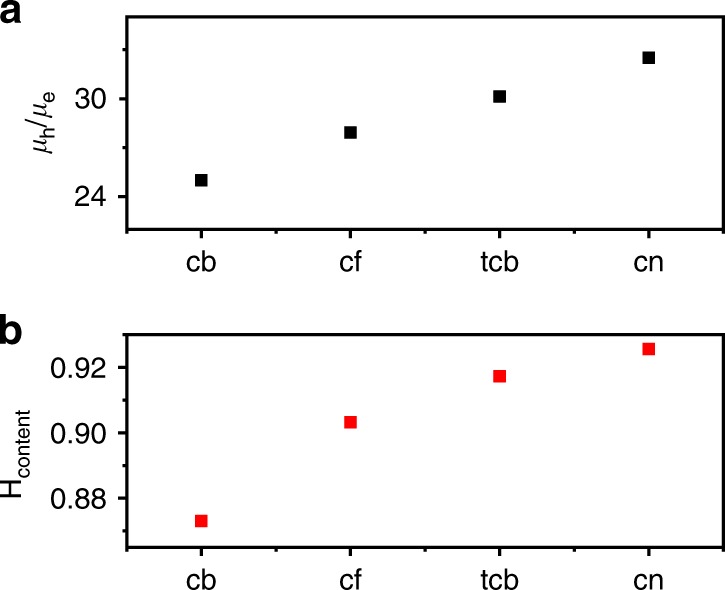
Solvent dependence of mobility and *I*_0–0_/*I*_0–1_. **a** Solvent dependence of the holes and electrons mobility ratio (*μ*_e_/*μ*_h_). **b** Solvent dependence of the relative peak ratio between the *I*_0–1_ and *I*_0–0_ peak (∝ *λ*^2^) found in A_//_ UV–Vis. Such ratio is proportional to the H-type electron-vibration coupling. Films spun from toluene are characterized by a negligible dichroic ratio and are not considered here. Trichlorobenzene (tcb), chloroform (cf), chlorobenzene (cb), chloronaphthalene (cn)

In Fig. 6 we compare the solvent dependence of the holes to electrons mobility ratio (*μ*_h_/*μ*_e_), with the H-type interactions content estimated from the *I*_0–0_/*I*_0–1_ value found in the *A*_⊥_ of each polymer film showing optical dichroism. The two plots in Fig. 6 clearly show the steady increase in *μ*_h_/*μ*_e_ with the strength of H-type coupling within the aligned film. Therefore, not only holes are selectively bleaching H-type interactions, but also the prevalence of H-type interactions at the ground state is shown here to be predictive of a more favored hole transport. At the same time, the presence of J-type interactions is predictive of a more favored electron transport in the polymer film.

Due to the rich amount of structural and charge transport investigations performed so far by different groups, it is possible to find ready available literature data to further confirm and generally extend the effectiveness of such ground-state analysis for predicting the semiconductors charge transport properties, also in unipolar polymer devices.

In their work, Chen et al.^68^ investigated the ambipolar properties of two polyselenophene derivatives. The relative prevalence of H- or J-type coupling signatures can be distinctly identified in both UV–Vis and PL spectra. From their data, an increasing H-type character observed in the ground state corresponds to a higher *μ*_h_/*μ*_e_ (Table 1), confirming our finding. In the same work, the authors also compare the same polyselenophene polymer backbone at varying the alkyl side chains group. Similarly to the previous observation, the different packing induced by the different side chains can promote either a relative increase in H- or J-type coupling content in the film. Also in this case, the correlation between H-type coupling content and hole mobility is present (Table 1). In a second work, Chen et al.^69^ observed, in the case of a polyselenophene homopolymer, comparable CMS spectral features to the one found in this work for *h-acc* and *e-acc*, showing a stronger J-type character in *e-acc* CMS. Such observation is valid also when comparing mobility values of different films of unipolar polymer devices. Interestingly, in the case of the n-type devices based on a well-known naphthalene-diimide copolymer (P(NDI2OD-T2)) investigated by D’Innocenzo et al.^70^, the effect of polymer film annealing on the OFET performance could have been predicted from the analysis of the ground-state spectra. The strong J-type character of P(NDI2OD-T2) is highlighted in its absorption spectra, coherently with its mostly favored electron transport properties in such device configuration. The electron mobility is higher for the pristine films, characterized by a stronger J-type character, than the melt-annealed ones (Table 1). Further examples showing the validity of our observation over different ambipolar and unipolar polymer devices, as well as homopolymers and D–A polymers, can be found briefly described in Supplementary Note 3. Our findings are therefore solid with respect to the existing literature where sufficient data have been provided to test its validity.

**Table 1:** Relating relative mobilities with the relative content of H-type and J-type coupling in ambipolar polymer (P3OS^69^, PSS-C10, PSS-C8, and PSS-C6^68^) and electron transport to J-type coupling content in unipolar polymer (P(NDI2OD-T2))^70^

Polymer semiconductor	H-type coupling (*I*_0–1_/I_0–0_)	J-type coupling (*I*_0–0_/*I*_0–1_)	*μ*_h_/*μ*_e_	*μ*_e_ (cm^2^ V^−1^ s^−1^)	Reference
**P3OS**	1.65		5		^69^
**PSS-C10**	1.14		2		^68^
**PSS-C8**	1.30		3		^68^
**PSS-C6**	1.75		27		^68^
**P(NDI2OD-T2) pristine**		1.29		0.056	^70^
**P(NDI2OD-T2) melt annealed**		1.10		0.041	^70^
**DPPT-TT**^**a**^ **(chlorobenzene)**	0.87		25		
**DPPT-TT**^**a**^ **(chloroform)**	0.90		28		
**DPPT-TT**^**a**^ **(trichlorobenzene)**	0.92		30		
**DPPT-TT**^**a**^ **(chloronaphthalene)**	0.93		32		

^**a**^Data from this work. *I*_0–1_/*I*_0–0_ is proportional to the Huang–Rhys factor

## Discussion

We reported a series of coherent experimental observations that demonstrate intrinsic charge selectivity for intra- or intermolecular interactions within the same semiconducting polymer film microstructure. The fine structure found in the CMS spectra confirms that both electrons and holes relax into ordered polymer aggregates. Both hole and electron transport are favored by the alignment of the backbone chains and are maximized along the direction of the conjugated polymer backbone. The effectiveness of the solid-state film in transporting both holes and electrons can be monitored from the degree of alignment (dichroic ratio) extracted from a simple absorption spectrum. In D–A copolymers, also the relative intensity of the CT state can be monitored to assess the increasing mobility by evaluating its relative weight in the ground-state absorption spectra.

Most importantly, we showed that the transport of either a hole or an electron is selectively assisted by the presence of H-type interactions or J-type interactions, respectively. Though the absolute value of the charge mobility is principally related to the presence of a polymer ordered structure, chain alignment, presence of static and dynamic disorder, and ground state with CT character, the prevalence of either hole or electron transport is a function of the relative content of H- and J-type interactions.

For hole transport, a strong perturbation of the H-type interactions occurs, as demonstrated by their optical bleaching. The correspondence between this selective perturbation and the increased hole mobility demonstrates that H-type intermolecular interactions mostly favor hole transport. Similarly, the increasing electron mobility with the increased J-type character of the ground-state optical properties shows that J-type intramolecular interactions favor electron transport.

The extensive consistency of our findings with previously reported data, collected both on D–A copolymers and homopolymers, remarks the general application of the proposed simple ground-state analysis for the prediction of the field-effect device performances. We speculate that there must be a general reason for such specificity of hole preference to accommodate into H-type configurations and of electrons into J-type ones. A tentative explanation can be provided following first-order electrostatic arguments. At the first order, we can expect that the minimum relative distance to which cofacial chromophores can approach each other is limited by the increasing electrostatic repulsion between their outer electron densities. Therefore, in the presence of a negative charge, the best compromise for reducing such electrostatic repulsion is to relax into J-type intramolecular interactions rather than into H-type ones. The opposite occurs if a positive charge populates the aggregate, as an H-type configuration would lead a positive polaron to more favorably couple with the negative electronic cloud of the neighboring and cofacial chromophores.

Overall, with this work we propose the possibility of screening polymer solution formulations and film-processing conditions toward optimal electron or hole transport properties, simply with ground-state optical measurements, thus drastically reducing the effort and the cost of a full device fabrication and characterization through the typical “trial and error” approach. Furthermore, the charge selectivity upon specific molecular interactions can introduce a new parameter to be included within the design principles so far followed for achieving the desired unipolarity and ambipolarity of organic semiconductor devices. From a polymer design point of view, this might suggest new strategies to infer an H- or J-type character through the proper choice of heteroatoms, aromatic core dimension, or side chain engineering. This study therefore unlocks a previously noninvestigated key aspect to boost even further the potential of organic semiconductor optoelectronics, fostering the development of analytical tools for drastically accelerating improvements in the field.

## Methods

### Aligned film preparation

The number-average molecular weight (Mn) of DPPT-TT is 70,000 g mol^−1^ with a polydispersity index of 3.13, as determined by using high-temperature gel-permeation chromatography (GPC) at 135 ˚C with 1,2,4-trichlorobenzene as the eluent. Solution concentration was 10 mg/mL in chlorobenzene. For the off-center spin coating, the clean Corning glass substrate or the one carrying the electrode pattern was located at ~2 cm from the spin-coater axis center (1500 rpm, 3-s acceleration, in a N_2_-purged glove box). The action of the centrifugal forces induced by the fast rotation of the spin coater determines strong fluid-dynamic forces that induce the polymer chains to align along the radial direction. Not-aligned films were obtained by placing the substrate at the center of the spin coater.

### Thin-film characterization

UV−Vis spectrometry was conducted by using a Cary Varian Eclipse spectrometer, and a polarized film was used in front of the beam source.

The DPPT-TT polymer films used for the ground-state optical investigation were spin-coated from chlorobenzene solutions (10 mg/ml) at 1000 rpm.

### Temperature-dependent optical transmission

Samples were prepared through spin coating from chlorobenzene solutions (10 mg/ml). Temperature-dependent optical transmission spectra were acquired with a homebuilt UV–Vis–NIR spectrometer with a lock-in-based technique. The setup consisted of a tungsten lamp source, a monochromator, a Si-diode detector (Thorlabs FDS100), transimpedance amplifier (Femto DHPCA-100), and a lock-in amplifier (Standford Instrument SR830) connected to a computer. A MATLAB software was used to run the measurements. The transmission signal was acquired by modulating the monochromatic light with an optical chopper (Stanford Instrument SR540). Typical transmission spectra were obtained by dividing the modulated transmission acquired on the film (*T*) with the modulated transmission acquired on the reference glass sample (*T*_0_). The absorption spectra were obtained from its typical relation to the transmission signal (–log(*T*/*T*_0_)). A continuous-flow static exchange gas cryostat (Oxford Instruments) was employed with cryogenic liquid (N_2_).

### Transient absorption (TA)

Samples for TA studies were prepared on a glass substrate following the spin-off-center coating procedure. A short pump pulse (∼220 fs, 530 or 855 nm) was used as the photoexcitation pump beam (fluence always comprised between 8.6 and 14 µJ/cm^2^), while the excited state dynamics were probed with a delayed broadband probe pulse. Transmission changes Δ*T* were measured as a function of the pump–probe delay and of probe wavelength. Τhe plotted signal is given by the differential transmission Δ*T*/*T* = [(*T*pump on –*T*pump off)/*T*pump off]. A mode-locked oscillator at 100 kHz (Pharos—Model PH1-20-0200-02-10, Light Conversion, Lithuania) emitting 1030-nm pulses was used to generate both pump and probe beams. Pump wavelengths were generated coupling 10 W from the oscillator into a commercial optical parametric amplifier (Orpheus, Light Conversion, Lithuania), while probe wavelengths in the spectral range 500–1050 focusing 2 W of the oscillator power onto a sapphire crystal generated nm. Pump and probe pulses were focused and spatially overlapped on the sample, ensuring that the spot size of the probe beam was significantly smaller with respect to the pump beam. The transmitted probe signal was coupled into an imaging spectrograph (Shamrock 193i, Andor Technology Ltd., UK), combined with a multichannel detector. All measurements were taken with the samples in a vacuum chamber to prevent any influence from oxygen or sample degradation.

### Field-effect transistor fabrication

Prepatterned Corning Eagle 2000 glass substrates were cleaned by sequential ultrasonication in deionized water, acetone, and isopropanol (15 min each). Source and drain electrodes were fabricated by using conventional photolithography (channel width W and channel length ratio, *W*/*L* = 1.0 mm/20 μm), followed by thermal evaporation of Ni (5 nm) and Au (15 nm). The substrates were treated with oxygen plasma for 20 min before spin-coating the polymer solution. DPPT-TT solutions for devices were prepared with a concentration of 10 mg/mL in chlorobenzene. The off-center spin coating was performed at 1500 rpm (3-s acceleration) in an N_2_-purged glovebox. The electrode pattern was placed 20 mm away from the rotation axis of the spin coater. The semiconductor layer thickness (30−50 nm) was measured with a surface profiler (Kosaka ET-3000i). The DPPT-TT film was thermally annealed at 200 °C for 20 min to remove the residual solvents under N_2_ atmosphere.

Polymethylmethacrylate (PMMA, Aldrich, Mw = 120 kDa) was used as dielectric material without further purification, at a concentration of 80 mg/mL in n-butylacetate. The solution was filtered with a 0.45-μm PTFE syringe filter before spin coating. After deposition, the devices were further annealed at 80 °C for 2 h under N_2_. The optically semitransparent gate electrode was thermally evaporated and consisted of 4-nm Al top-gate electrode and 8-nm Au.

### Device characterizations

The OFET electrical characteristics were measured by using a semiconductor parameter analyzer (Agilent B1500A) in an N_2_-filled glovebox on a Wentworth Laboratories probe station. The field-effect mobility (μFET) was calculated from the saturation regime by using equations for classical silicon MOSFETs as previously reported.

### Charge-modulation spectroscopy

All macroscopic CMS measurements are performed with a homemade vacuum chamber (~10^−5^  mbar) in a transmission configuration. Source and drain electrodes were kept grounded, while the gate bias modulation was generated by a waveform generator (Keithley 3390) and a voltage amplifier (Falco Systems WMA-300). A tungsten lamp was employed as light source along with a monochromator. The light was focused on the device and the transmitted light signal was recorded with silicon photodetector (Thorlabs FDS100). The signal was then amplified with a transimpedance amplifier (Femto DHPCA-100) and then sent to a lock-in amplifier (Standford Instrument SR830) to obtain the differential transmission signal, Δ*T*.

### Polarized charge-modulation microscopy

The p-CMM data were collected with a homemade confocal microscope that operated in transmission mode. The light source consisted of a supercontinuum laser (NKT Photonics, SuperK Extreme) monochromated by an acousto-optic modulator (NKT Photonics, SuperK Select) in the 500−1000-nm region with line widths between 2 and 5 nm. Laser polarization was controlled with a half-wave plate and linear polarizer. The light was then focused on the sample with 0.7 N.A. objective (S Plan Fluor60x, Nikon) and collected by a second 0.75 N.A. objective (CFI Plan Apochromat VC ×20, Nikon). The collected light was focused on the entrance of a multimodal glass fiber with 50-μm core that acted as confocal aperture. Detection was operated through a silicon photodetector (FDS100, Thorlabs). The intensity of the collected light was registered by a silicon photodetector (Thorlabs FDS100). The signal was amplified by a transimpedance amplifier (DHPCA-100, Femto) and supplied both to DAQ (to record the transmission signal, *T*) and a lock-in amplifier (SR830 DSP, Stanford Research Systems) in order to retrieve the differential transmission data, Δ*T*. The charge-modulation signal is obtained by dividing the differential transmission signal, to transmission signal (Δ*T*/*T*). A custom Labview program was used to run the measurements for data collection and to interface the laser source and a 3D piezo stage (Physik Instrumente P-517). The same software control of the piezoelectric element was used to perform local CMS spectra in a specific location within the OFET channel. Samples were placed in a homemade chamber with electrical feedthroughs to keep the sample under a continuous flow of nitrogen.

The voltage modulation at gate electrode is obtained by amplifying the voltage signal from the waveform generator (Keithley 3390) with a voltage amplifier (Falco Systems WMA-300). The gate modulation frequency was *f* *=* 993 Hz unless otherwise specified. Prior to each measurement, the phase of the lock-in amplifier was synchronized to the amplified voltage modulation signal from the voltage amplifier at the first harmonic detection of the lock-in amplifier. A dual channel source-measure unit (Agilent B2912A) was used to keep the drain and source at ground.

## Supplementary information

Supplementary Information

## Data Availability

Relevant data generated or analyzed during this study are included in this published article (and its supplementary information files). Any further source data are available from the corresponding author upon reasonable request.
