# Supplementary material for: Intrinsically distinct hole and electron transport in conjugated polymers controlled by intra and intermolecular interactions
Source: Nat Commun. 2019 Nov 19;10:5226. doi: 10.1038/s41467-019-13155-9 (PMC6863910; doi:10.1038/s41467-019-13155-9)
Supplement: Supplementary file 1 — Supplementary Information [file 41467_2019_13155_MOESM1_ESM.pdf]

Supplementary Information

Intrinsically distinct hole and electron transport in conjugated polymers role of intra and intermolecular interactions

Giuseppina Pace,[1,*] Ilaria Bargigia,[1,2] Yong-Young Noh,[3] Carlos Silva, [2] Mario Caironi[1,*]

[1] *Center for Nano Science and Technology@PoliMi, Istituto Italiano di Tecnologia, Via Pascoli 70/3, 20133 Milano, Italy*

[2] *School of Physics, Georgia Institute of Technology, Atlanta, Georgia, USA*

[3] *Department of Chemical Engineering, Pohang University of Science and Technology, 77 Cheongam-Ro, Nam-Gu, Pohang 37673, Republic of Korea*

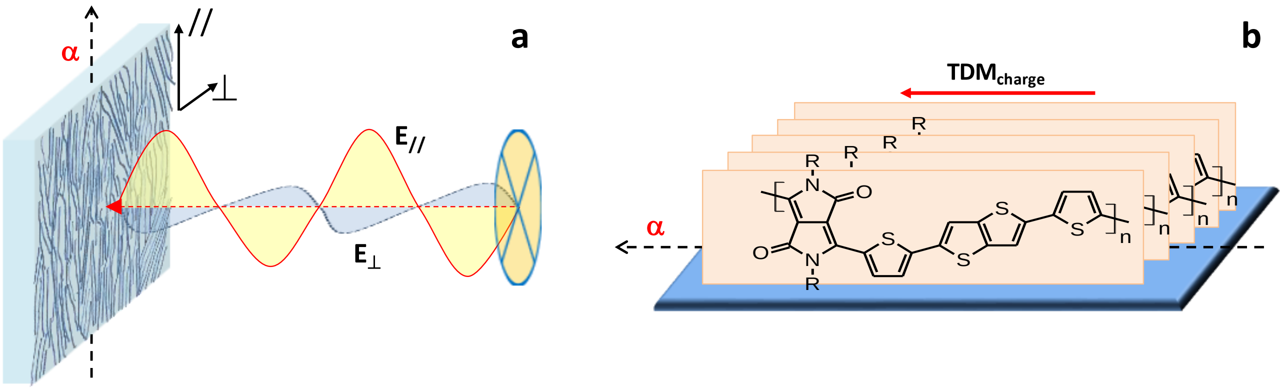

Supplementary Figure 1: Sketch of an aligned polymer film. a) Aligned polymer film under an incident polarized light beam whose electromagnetic field is either perpendicular ($E_{\perp}$) or parallel ($E_{//}$) to the polymer chain alignment direction (α). b) Qualitative sketch of the edge-on configuration of the polymer chains at the top surface of the polymer film as found with 2D-GIXRD. The charge transition dipole moment (TDM_{charge}) orientation relative to the polymer chain alignment is also shown.

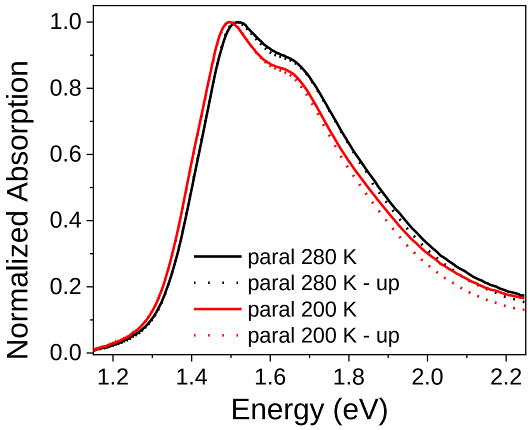

Supplementary Figure 2: Temperature dependent UV-Vis. The figure show the reversible absorption spectra acquired during the cycling of the temperature from 280 K down to 200K and up again to 200 K (up) and 280 K (up).

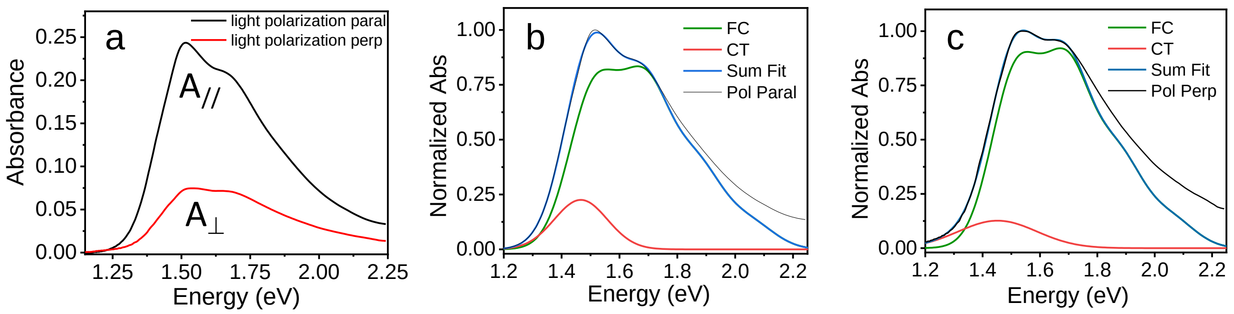

Supplementary Figure 3: Franck-Condon analysis (FC) of spectra reported in Figure 1a of the main text. a) Polarized UV-Vis absorption spectra: Light polarized parallel (polar paral, $A_{//}$) or perpendicular to the chain alignment direction (polar perp, $A_{\perp}$). FC of the Uv-Vis spectra acquired under incident parallel (b) and perpendicular polarized light (c). The Sum Fit spectrum is obtained from the sum of the FC curve fit of the main π-π optical transition and the CT (Gaussian) peak. A more prominent contribution of the CT peak is present in $A_{//}$ (b) in agreement with the TA findings.

Supplementary Note 1. Franck-Condon analysis. In **Supplementary Figure 3** we report a Franck-Condon analysis of the absorption spectra reported in Figure 1a of the main text. The Linear absorption data were fitted using a Frank Condon progression considering four vibronic levels and with the Huang Rhys factor, the energy of the 0-0 transition, the width of the gaussian, and a proportionality constant as free parameters. The energy of the C=C stretching mode was fixed at 0.18 eV. This analysis is not intended to be an accurate and rigorous study of the absorption peaks, which instead would require a more extensive theoretical work. The complexity of the system rely on the coexisting inter- and intrachain interaction and their spectral convolution with the CT state, whose contribution to the spectrum would require the difficult task to define a proper interaction Hamiltonian (Chem. Rev. 2018, 118, 7069−7163, Acc. Chem. Res. 2017, 50, 341−350). Nevertheless, we can demonstrate that the best fit of the spectra is obtained with a combination of a Franck-Condon (FC) fit for the π-π interaction and a Gaussian function for the red shoulder.

The FC progression perfectly fits the vibronic structure presents in the π-π transition, where a peak to peak distance of 0.18 eV is found between the vibronic peaks (I_{00}, I_{01}, I_{02}) and which falls into the vibrational region of the diketopyrrolepyrrole (DPP) unit C=C stretching mode (J. Mater. Chem. C, 2017, 5, 6176-6184). The red shoulder is instead spaced at 0.05-0.07 eV distance from the I_{00} vibronic peak, showing that it does not belong to the same vibronic progression.

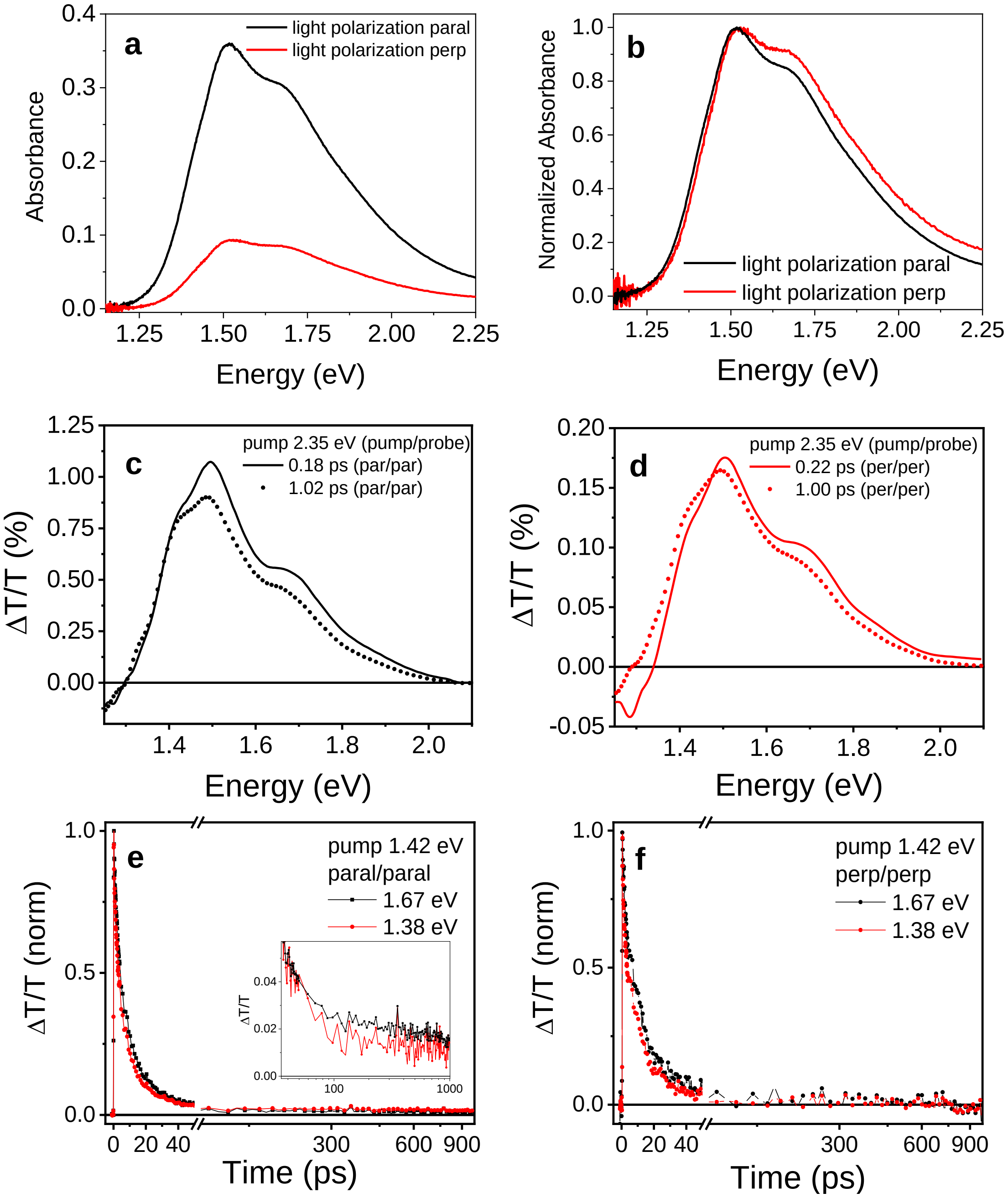

Supplementary Figure 4: UV-Vis and Transient Absorption (TA) spectra. a-b) Uv-Vis absorption spectra acquired under perpendicular and parallel incident light polarization; b- normalized spectra. c-d) TA spectra acquired at a pump beam 2.35 eV at varying the probe beam delay (~0.2 ps and ~ 1ps) showing the faster decay (~ 10 ps) characterizing the blue part of the spectra; e-f) Time decay of TA signal acquired at a pump beam of 1.42 eV with pump/probe polarization paral/paral (e) and perp/perp (f). The longer lived dynamic (up to 1 ns) which has been assigned to the CT ground state repopulation is clearly visible for pump and probe beams polarized parallel to the polymer chain (panel e), while a fast decay is present for the perp/perp (pump/probe, panel f). These data confirm the alignment of the CT transition dipole moment along the polymer chain. Film prepared with the spin-off center coating and from 10 mg/ml chloronapthalene solution followed by annealing at 200°C).

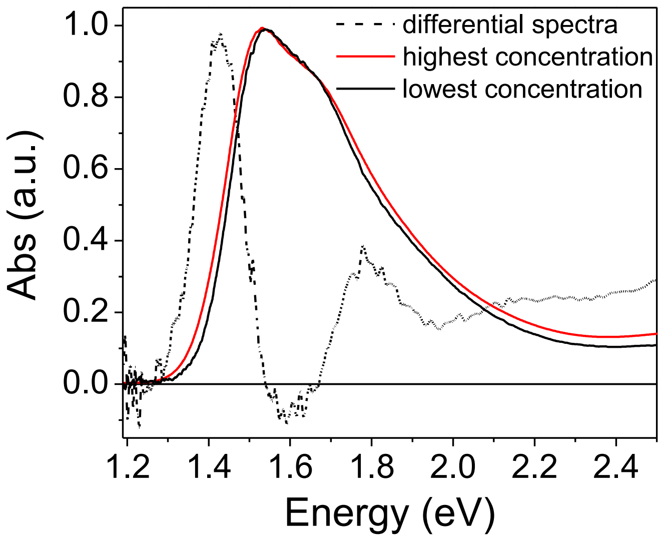

Supplementary Figure 5: UV-Vis absorption spectra acquired in solution. DPPT-TT solutions in chlorobenzene at the lower concentration (1.5 x 10^{-6} M, red line) and at the higher concentration (17.5 x 10^{-6} M, black line) showing the different absorption features of a non-aggregate vs an aggregated polymer chain. The dotted line shows the differential spectra highlighting the red shoulder prominence in the aggregate spectra appearing at 1.45 eV.

Supplementary Table 1: I_{0-0}/I_{1-0} ratio in solution and film. Peak position associated to the absorption spectra acquired on DPPT-TT in solution and films. I_{1-0} peak values were extracted from the second derivative of the absorption.

	**I_{0-0} eV (Intensity value)**	**I_{1-0} eV (Intensity value)**	**I_{0-0}/I_{1-0}**
**Diluted solution**			1.24
parallel component (fig 1a)	1.512 (1)	1.658 (0.837)	1.19
orthogonal component (fig 1a)	1.518 (1)	1.651(0.923)	1.08
parallel component 80 K (fig 1b)	1.487 (1)	1.658 (0.813)	1.23
orthogonal component 80 K (fig 1b)	1.494 (1)	1.662 (0.828)	1.20
parallel component RT (fig 1b)	1.518 (1)	1.685 (0837)	1.19
orthogonal component RT (fig 1b)	1.535 (1)	1.685 (0944)	1.06
**CMS *h-acc* local spectra**	1.514 (4.3 x 10^{-4})	1.68 (3.4 x 10^{-4})	1.26
**CMS *e-acc* local spectra**	1.512 (4.4 x 10^{-4})	1.67 (3.1 x 10^{-4})	1.42

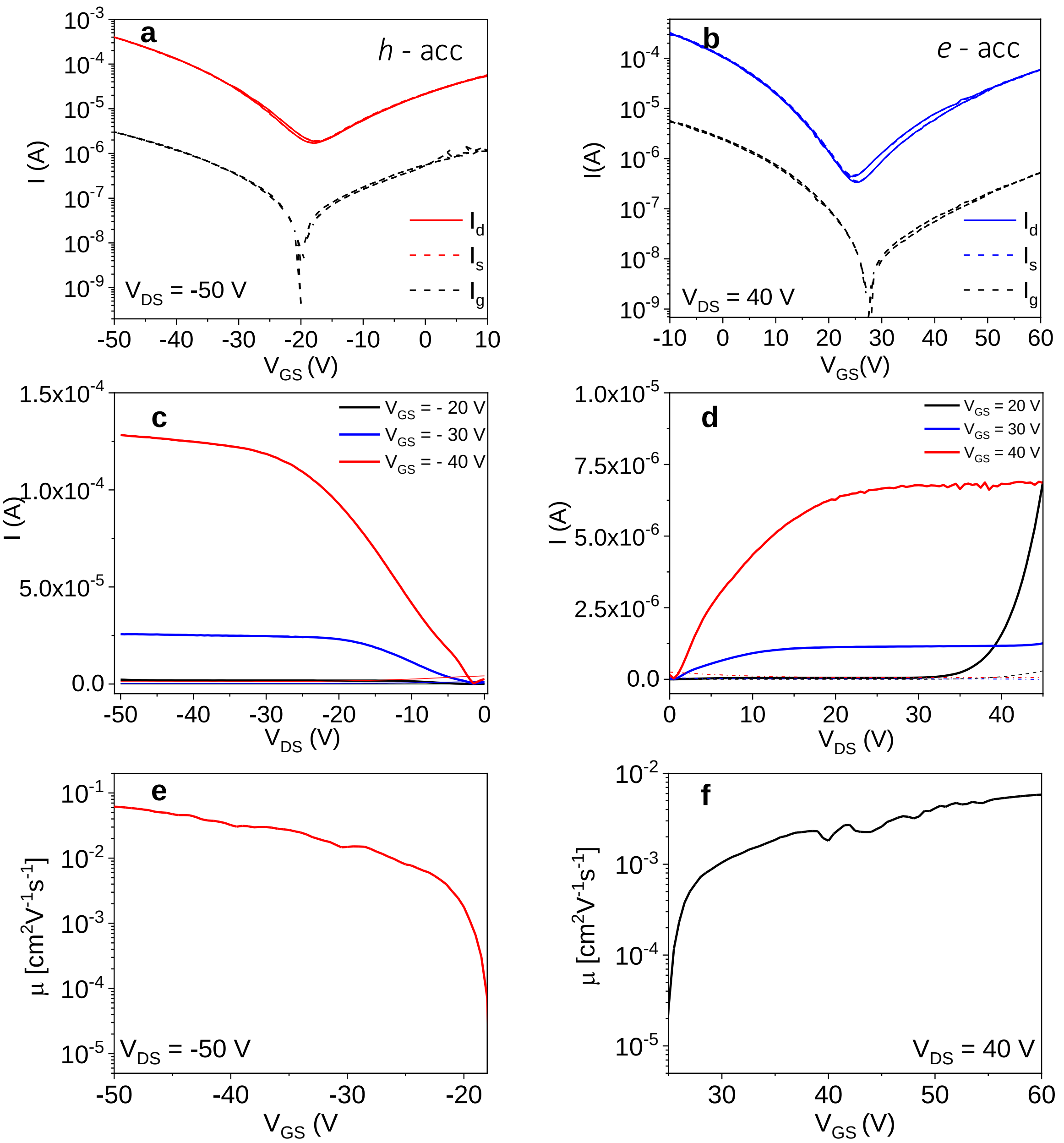

Supplementary Figure 6: Electrical characteristics of DPPT-TT FET. The polymer chains alignement is perpendicular to the channel length: a) and b) transfer curves; c) and d) output curves. e- holes and f- electrons mobility acquired in saturation regimes (channel width W, channel length ; W/L = 1.0 mm/20 μm; PMMA gate dielectric thickness 500 nm and capacitance ~6.2 nF/cm^2). The mobilities dependence from V_{GS} in saturation regime, were calculated from the slope of I_{drain} vs V_{GS} ($\sqrt{I_{drain}}$ vs V_{GS}) according to the gradual channel approximation. For a more comprehensive study of the mobility dependence, please refer to Kim *et al.*[2]

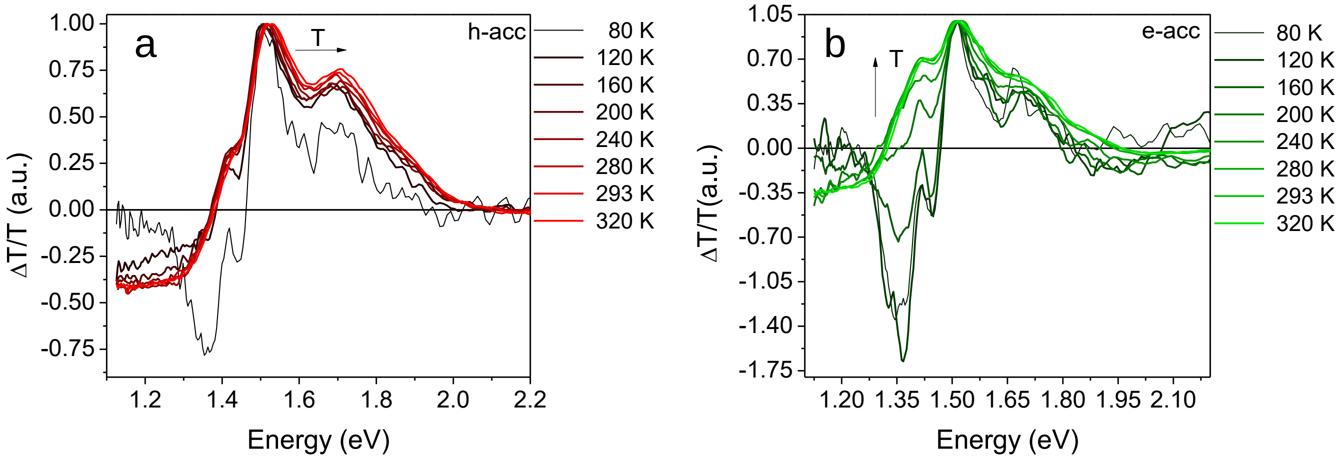

Supplementary Figure 7: Normalized temperature dependent CMS. Spectra acquired under holes accumulation a) and electrons accumulation (b) regimes. The contribution of electroabsorption starts being more visible at T lower than 150 K for e-acc regimes and at even lower T for h-acc. (*e-acc*: V_g= + 30 V; V_{pp} = 40 V ± 20 V); *h-acc*: V_g= - 30 V; V_{pp} = 40 V ± 20 V).

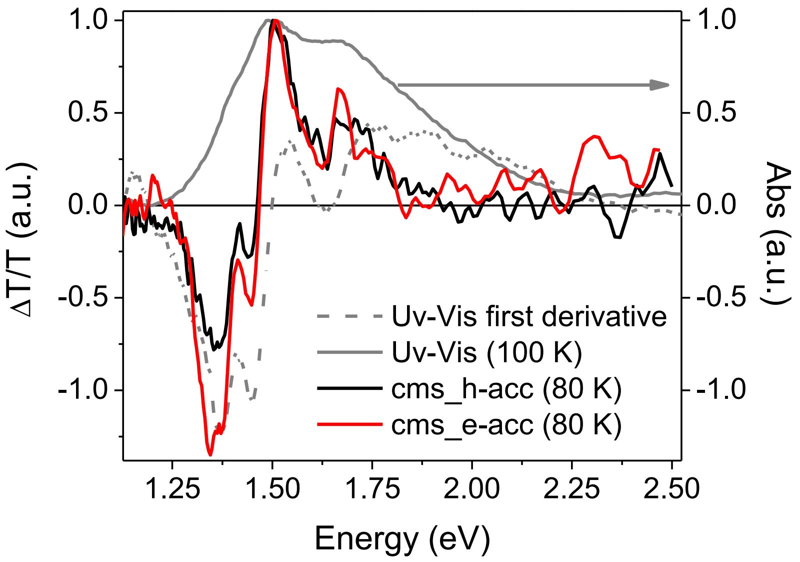

Supplementary Figure 8: Comparing e-acc and h-acc spectra at low temperature (80 K). In the absence of charge accumulation in the OFET the spectra resemble the first derivative of the J-like absorption spectra as expected. At low temperature (< 100 K) where no charge accumulation is occurring due to the increased injection barrier, the CMS spectra is mostly dominated by the electroabsorption (EA) features. We already observed that at low temperature, the planarization of the polymer backbone leads to an increase of J-like features, therefore, we observe the e-acc and h-acc spectra to converge to similar EA features.

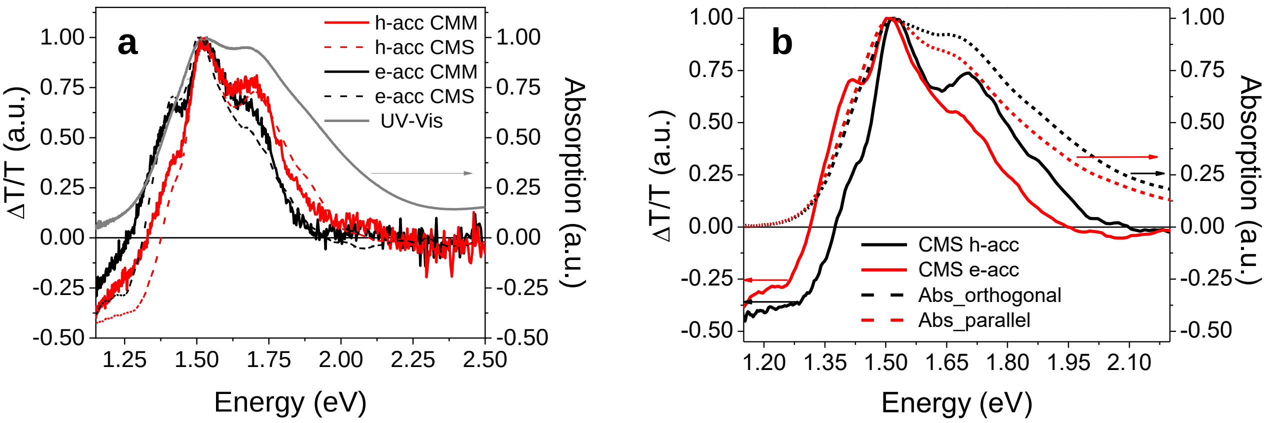

Supplementary Figure 9: Macroscopic versus local CMS. a- Comparison between charge modulation spectra acquired under h-acc and e-acc either locally within the active channels (CMM) or over the entire device area (CMS). b- Normalized room temperature CMS spectra overlapped with the parallel and orthogonal anisotropic components of the absorption spectra.

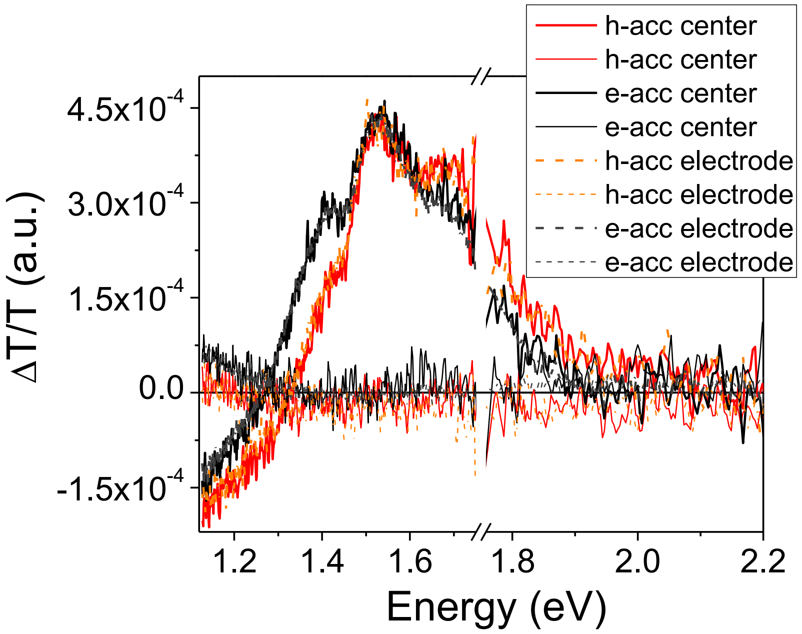

Supplementary Figure 10: **Local CMS within the FET channel and on electrodes.** No difference is observed in the local CMS spectra acquired either in the channel center, or at the electrode edge, implying the presence of low charge injection barrier at room temperature and the absence of spurious interferences arising from the gold electrodes. Out-off phase lock-in signal is also reported per each accumulation regime. (Gold electrodes are ~ 20 nm thick to enable optical transparency).

Supplementary Note 2: Transition dipole moment (*TDM*$_{\text{charge}}$) and degree of order (*DO*). *TDM*$_{\text{charge}}$ (*TDM*) and *DO* maps are acquired at six different laser polarization angles θ. The intensity of the charge modulation signal (CM) found per each image pixel coordinate (x,y) is defined as $I_{(x,y)}(\theta)$. Data are fitted according to the following equation[1]:

(1)
$$I_{(x,y)}(\theta) = M_{(x,y)} \cos^2\left(\beta_{(x,y)} - \theta\right) + C_{(x,y)}$$

where $C_{(x,y)}$ is the fraction of signal independent from the laser polarization and is related to the randomly distribute *TDM*. $M_{(x,y)}$ is the amplitude of the polarization dependent CM signal, and $\beta_{(x,y)}$ is the *TDM* preferential alignment direction.

From the extracted $M_{(x,y)}$ and $C_{(x,y)}$ quantitative information on the fraction of aligned *TDM* can be derived, according to the following equation:

(2)
$$DO_{(x,y)} = \frac{M_{(x,y)}}{M_{(x,y)} + 2C_{(x,y)}}$$

Factor 2 takes into account that anisotropic *TDM* has halved probability, on average, to absorb a photon. The resulting *DO* map indicates the fraction of CM signal arising from anisotropically distributed *TDM*.

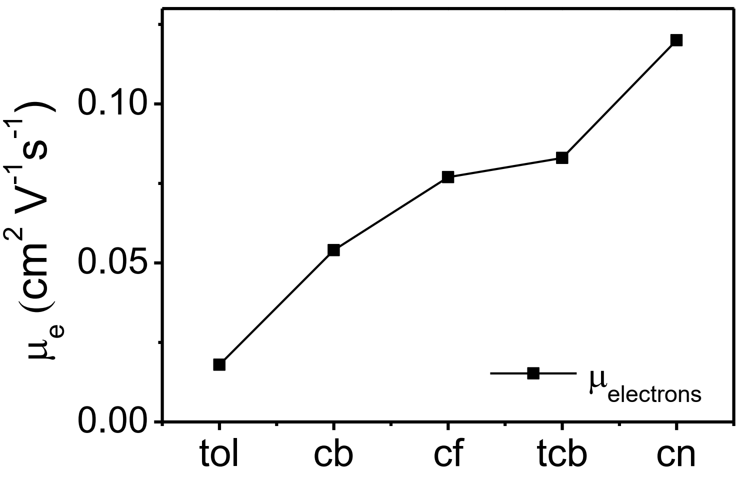

Supplementary Figure 11. Solvent dependent electron mobility. OFETs are prepared from off-center spin coated films. Polymer chains are aligned perpendicularly to the Source and Drain electrodes. The increasing mobility follows the increasing content of the CT ground state as found also for holes mobility.

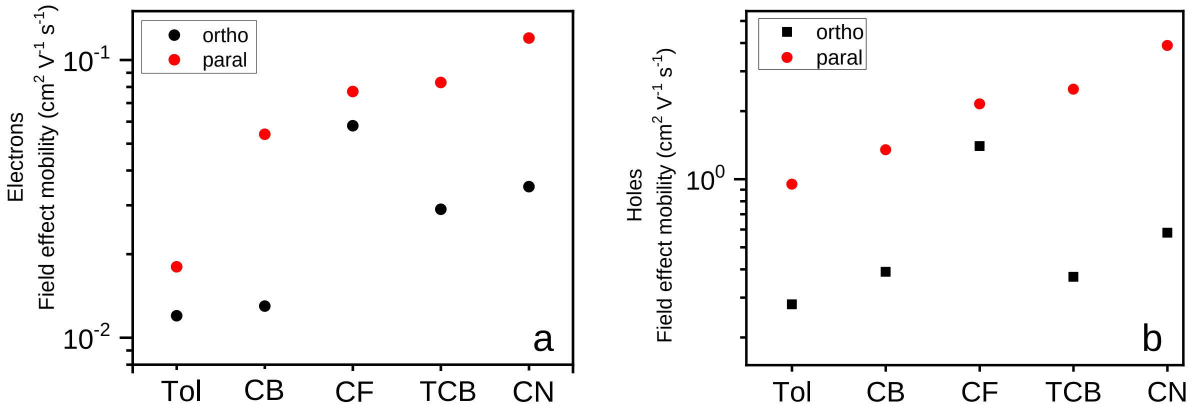

Supplementary Figure 12: Comparing the solvent dependence of the electron (a) and hole (b) mobility. Polymer chains in the devices were aligned either orthogonal or parallel to the source and drain electrodes. Data extracted as in reference 2 and 3.[2,3]

Supplementary Note 3. Further examples of the mobility dependence upon J (H) character. The following examples are taken from the literature and support the main conclusion of this work.

Adv. Mater. ***2018****, 30, 1704843*. In this paper a very pronounced J character can be observed in all the new polymers presented by the authors, and as expected all the reported polymers have a pronounced n-transport (e.g. N2200).

Macromolecules, **2006**, *39* (25), 8712. The authors present a series of conjugated thieno[3,4-b]pyrazine-based donor-acceptor copolymers at varying acceptor and/or side groups. The absorption spectra reported in Figure 3 of the cited paper show polymers with more pronounced H-character that well aligning with the observed hole transport for all polymers. Furthermore, a better resolved H-type vibronic structure is observed for the BTTP-F polymer followed by the BTTP-P and BTTP one. Table 1 reported by the authors shows how the highest hole mobility is indeed found for the BTTP-F polymer.

Supplementary References

1 Martino, N. *et al.* Mapping Orientational Order of Charge-Probed Domains in a Semiconducting Polymer. *Acs Nano* **8**, 5968-5978 (2014).

2 Kim, N. K. *et al.* High-Performance Organic Field-Effect Transistors with Directionally Aligned Conjugated Polymer Film Deposited from Pre-Aggregated Solution. *Chem Mater* **27**, 8345-8353 (2015).

3 Kim, N.-K., Shin, E.-S., Noh, Y.-Y. & Kim, D.-Y. A selection rule of solvent for highly aligned diketopyrrolopyrrole-based conjugated polymer film for high performance organic field-effect transistors. *Org Electron* **55**, 6-14 (2018).
